# Composite Multiscale Partial Cross-Sample Entropy Analysis for Quantifying Intrinsic Similarity of Two Time Series Affected by Common External Factors

**DOI:** 10.3390/e22091003

**Published:** 2020-09-08

**Authors:** Baogen Li, Guosheng Han, Shan Jiang, Zuguo Yu

**Affiliations:** 1 Key Laboratory of Intelligent Computing and Information Processing of Ministry of Education and Hunan Key Laboratory for Computation and Simulation in Science and Engineering, Xiangtan University, Xiangtan 411105, China; libaogen@xtu.edu.cn (B.L.); hangs@xtu.edu.cn (G.H.); 201831510086@smail.xtu.edu.cn (S.J.); 2 School of Electrical Engineering and Computer Science, Queensland University of Technology, GPO Box 2434, Brisbane QLD 4000, Australia

**Keywords:** composite multiscale partial cross-sample entropy (CMPCSE), multiscale cross-sample entropy (MCSE), time series, stock indices

## Abstract

In this paper, we propose a new cross-sample entropy, namely the composite multiscale partial cross-sample entropy (CMPCSE), for quantifying the intrinsic similarity of two time series affected by common external factors. First, in order to test the validity of CMPCSE, we apply it to three sets of artificial data. Experimental results show that CMPCSE can accurately measure the intrinsic cross-sample entropy of two simultaneously recorded time series by removing the effects from the third time series. Then CMPCSE is employed to investigate the partial cross-sample entropy of Shanghai securities composite index (SSEC) and Shenzhen Stock Exchange Component Index (SZSE) by eliminating the effect of Hang Seng Index (HSI). Compared with the composite multiscale cross-sample entropy, the results obtained by CMPCSE show that SSEC and SZSE have stronger similarity. We believe that CMPCSE is an effective tool to study intrinsic similarity of two time series.

## 1. Introduction

Complex systems with interacting constituents exist in all aspects of nature and society, such as geophysics [1], solid state physics, climate system, ecosystem, financial system [2,3], and so forth. These complex systems are constantly generating a large number of time signals. Fortunately, in recent decades, numerous creative methods have been proposed to explore the operation mechanism of these complex systems. Among them, entropy-based methods are very powerful modern analysis technology. The concept of ’entropy’ was first proposed by Clausius to deal with thermodynamic problems, and then Boltzmann gave a microscopic explanation from the perspective of statistical mechanics and proposed Boltzmann entropy. Gibbs proposed Gibbs entropy when determining uncertain system. In 1948, Shannon introduced the concept of entropy into information theory and put forward Shannon entropy (information entropy) [4]. Shortly after that, Renyi extended it and proposed Renyi entropy [5]. In 1988, Tsallis gave a Generalization of Boltzmann-Gibbs Statistics and proposed Tsallis entropy [6]. Although Gibbs entropy and Shannon entropy have the same mathematical expression, Shannon entropy has a broader meaning than thermodynamic entropy, as all the basic laws of thermodynamics can be derived from information entropy [7]. Since information entropy and Shannon entropy were proposed, many entropy-based methods have been proposed to explore the system complexity through studying the time series generated from them [8,9]. In order to quantify the changing complexity of real finite time series, Picnus proposed the approximate entropy (ApEn) [10,11,12], which had been used to study biological time series [13,14]. In 2002, Richman et al. analyzed the deficiencies of ApEn and proposed the concept of sample entropy (SampEn). Compared with ApEn, SampEn agreed with theoretical results much closer than ApEn over a broad range of conditions, and has been successfully applied to clinical cardiovascular study [15,16]. Cross-sample entropy (Cross-SampEn) was also proposed for comparing two different time series to assess their degree of similarity [15]. And in 2010, when Liu et al. studied the correlation of foreign exchange time series, they found that cross-SampEn is superior to correlation coefficient in describing the correlation between the foreign exchange time series [17]. In 2003, Costa et al. found that an increase in the entropy of a system is usually but not always associated with an increase of complexity, so the traditional entropy-based algorithms may lead to misleading results [18]. And in order to avoid this situation, they introduced the multiscale sample entropy (MSE), which had been successfully used to study various dynamical systems [19,20,21,22,23]. Not long after that, MSE was extended to multiscale cross-sample entropy (MCSE) to measure the cross-sample entropy over different time scales. Unfortunately, in the process of multi-scale analysis, the coarse-grained procedure sets a higher requirement for the length of the time series, that is, when the length of the sequence is not long enough, it will get inaccurate results. In addition, in some cases, the insufficiency of sequence length will lead to no template vector matched to another, and hence the cross-sample entropy can not be defined. In order to overcome this shortcoming, Wu et al. proposed the composite multiscale sample entropy (CMSE) [24] and refined composite multiscale entropy (RCMSE) [25] successively. Inspired by CMSE and RCMSE, Yin et al. introduced composite multiscale cross-sample entropy (CMCSE) and Refined composite multiscale cross-sample entropy (RCMCSE) [26], which reduced the probability of undefined entropy and has been successfully used to study structural health monitoring system [27]. In 2018, in order to better study the time series from the stock market, Wu and his coworkers introduced modified multiscale sample entropy measure based on symbolic representation and similarity (MSEBSS) [28]. Recently, Wang et al. proposed multiscale cross-trend sample entropy (MCTSE) to study the similarity of two time series that with potential trends [29]. In addition, multivariate multiscale sample entropy algorithm has been proposed to deal with multivariate data [30,31,32]. Recently, Jamin and Humeau-Heurtier offered a state-of-the-art on cross-entropy measures and their multiscale approaches in [33].

On the other hand, when some scholars studied the long-range correlation between time series, they found that if two non-stationary time series are driven by a common third-party force or by common external factors, the result without considering the common third-party force may not reflect their intrinsic relationship [34,35,36]. Fortunately, Baba et al. [37] found that if two time series affected by the external factors are additive, the levels of intrinsic cross-correlation between two time series can be measured by the partial cross-correlation coefficient. In 2015, Yuan et al. [38] and Qian et al. [39] introduced partial cross-correlation analysis to deal with this kind of situation from different departure points.

Inspired by the above works, we propose the composite multiscale partial cross-sample entropy (CMPCSE) to measure the intrinsic similarity of two time series affected by the third common external factor simultaneously in this paper. We first test CMPCSE on three sets of artificial data, and find that it can reveal the intrinsic similarity of the time series come from the models, and then apply it to a set of stock market indices.

## 2. Composite Multiscale Partial Cross-Sample Entropy

In this section, based on CMCSE [26], we propose a new method-composite multiscale partial cross-sample entropy (CMPCSE), which can be used to quantify the intrinsic similarity of two time series linearly affected by a common external factor.

Consider two time series recorded simultaneously, {x(t):t=1,2,…,N} and {y(t):t=1,2,…,N} linearly affected by {z(t):t=1,2,…,N}, the main steps of CMPCSE are as follows:

**Step 1:** First we eliminate the effect of z(t) on *x* and *y*, respectively. The additive model for models of x(t) and y(t) can be given respectively as:

(1) $$\left\{\begin{array}{c} x\left(t\right)=\beta_{x,0}+\beta_{x,1}z\left(t\right)+r_{x}\left(t\right),\\ y\left(t\right)=\beta_{y,0}+\beta_{y,1}z\left(t\right)+r_{y}\left(t\right), \end{array}\right.$$

where, t=1,2,…,N. When using regression analysis to estimate the value $r_{x}\left(t\right),r_{y}\left(t\right)$, in a window of length *s*, we use the idea in MF-TWXDFA [40] to remove the effect of the sequence z(t) on x(t) and y(t) point by point as follows. For a given integer *s* (s≥2), the points *j* contained in a sliding window $MW_{i}$ corresponding to the point *i* should satisfy |i−j|≤s. When the length of time series is different, we take different value for *s*. Usually the value of *s* is determined by experience. Accordingly, the weight function of the geographic weighted regression model is:

(2) $$\omega_{ij}=\left\{\begin{array}{cc} {\left[1-{\left(\frac{\left(i-j\right)}{s}\right)}^{2}\right]}^{2}, & if\;|i-j|\leq s,\\ 0, & otherwise. \end{array}\right.$$

In the window $MW_{i}$, we perform linear regression for $\left\{\omega_{ij}x_{j}\right\}$ on $\left\{z_{j}\right\}$ or $\left\{\omega_{ij}y_{j}\right\}$ on $\left\{z_{j}\right\}$, respectively. We can get the regression values $\hat{x}\left(z_{i}\right)$ and $\hat{y}\left(z_{i}\right)$ of x(i) and y(i), respectively. Then we get the corresponding estimates of $r_{x}\left(t\right),r_{y}\left(t\right)$:

$$\begin{array}{c} {\hat{r}}_{x}\left(i\right)=x\left(i\right)-\hat{x}\left(z_{i}\right)\\ {\hat{r}}_{y}\left(i\right)=y\left(i\right)-\hat{y}\left(z_{i}\right). \end{array}$$

Then the normalized data of ${\hat{r}}_{x}\left(t\right),{\hat{r}}_{y}\left(t\right)$ are defined as ${\bar{r}}_{x}\left(t\right)=\left({\hat{r}}_{x}\left(t\right)-<{\hat{r}}_{x}\left(t\right)>\right)/\delta_{{\hat{r}}_{x}\left(t\right)}$ and ${\bar{r}}_{y}\left(t\right)=\left({\hat{r}}_{y}\left(t\right)-<{\hat{r}}_{y}\left(t\right)>\right)/\delta_{{\hat{r}}_{y}\left(t\right)}$, respectively. Here <.> and δ are the corresponding mean and standard deviation. Next, we calculate the CMCSE of ${\bar{r}}_{x}\left(t\right)$ and ${\bar{r}}_{y}\left(t\right)$.

**Step 2:** Construct coarse-grained time series from the series ${\bar{r}}_{x}\left(t\right)$ and ${\bar{r}}_{y}\left(t\right)$ with the scale factor τ, respectively. Then we get $\left\{u_{k}^{\tau }\left(t\right)\right\}$ and $\left\{v_{k}^{\tau }\left(t\right)\right\}$. Each point of the ***k***-th coarse-grained time series at a scale factor of τ is defined as

(3) $$u_{k}^{\tau }\left(j\right)=1/\tau \sum_{i=(j-1)\tau +k}^{j\tau +k-1}{\bar{r}}_{x}\left(i\right),\;\;\;\;1\leq j\leq \left(N-k+1\right)/\tau ,1\leq k\leq \tau .$$

(4) $$v_{k}^{\tau }\left(j\right)=1/\tau \sum_{i=(j-1)\tau +k}^{j\tau +k-1}{\bar{r}}_{y}\left(i\right),\;\;\;\;1\leq j\leq \left(N-k+1\right)/\tau ,1\leq k\leq \tau .$$

**Figure 1:**
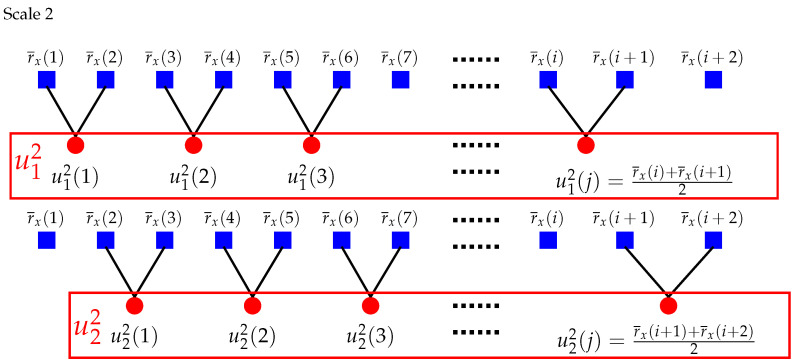
Schematic illustration of the coarse-grained procedure of composite multiscale partial cross-sample entropy (CMPCSE) when τ=2. Modified from Reference [24].

**Figure 2:**
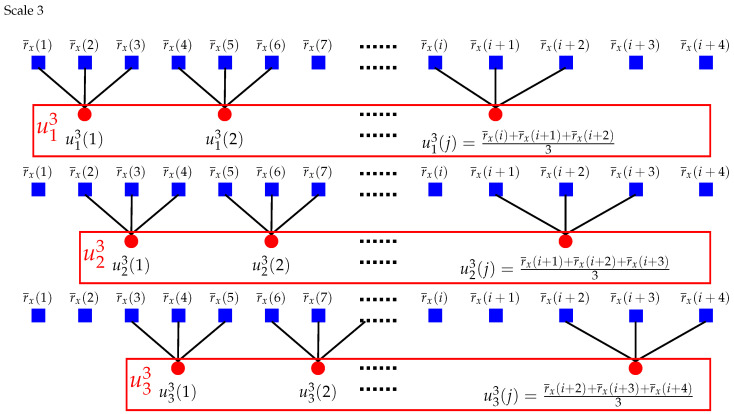
Schematic illustration of the coarse-grained procedure of CMPCSE when τ=3. Modified from Reference [24].

For scale one (τ=1), the times series $u_{1}^{1}$ and $v_{1}^{1}$ are the original series ${\bar{r}}_{x}$ and ${\bar{r}}_{y}$. For τ>1, Figure 1 and Figure 2 show two more intuitive examples of the coarse-grained procedure.

**Step 3:** According to the following formula, construct vector sequences with length *m*

(5) $${}^{m}h_{k}^{\tau }\left(i\right)=\left(u_{k}^{\tau }\left(i\right),u_{k}^{\tau }\left(i+1\right),\ldots ,u_{k}^{\tau }\left(i+m-1\right)\right),\;\;\;\left\{i:1\leq i\leq \left(N-k+1\right)/\tau -m+1\right\},$$

(6) $${}^{m}w_{k}^{\tau }\left(j\right)=\left(v_{k}^{\tau }\left(j\right),v_{k}^{\tau }\left(j+1\right),\ldots ,u_{k}^{\tau }\left(j+m-1\right)\right),\;\;\;\left\{j:1\leq j\leq \left(N-k+1\right)/\tau -m+1\right\},$$

from $\left\{u_{k}^{\tau }\left(t\right)\right\}$ and $\left\{v_{k}^{\tau }\left(t\right)\right\}$ respectively. Let ${}^{m}n_{k}^{\tau }\left(i\right)$ be the number of vectors ${}^{m}w_{k}^{\tau }\left(j\right)$ whose distance with ${}^{m}h_{k}^{\tau }\left(i\right)$

(7) $$d\left({}^{m}h_{k}^{\tau }\left(i\right),{}^{m}w_{k}^{\tau }\left(j\right)\right)=max\{|u_{k}^{\tau }\left(i+t\right)-v_{k}^{\tau }\left(j+t\right)\left|:0\leq t\leq m-1\right\}$$

is within the tolerance *r*. And then ${}^{m}n_{k}^{\tau }=\sum_{i}\;{}^{m}n_{k}^{\tau }\left(i\right)$ represents the total number of *m*-dimensional matched vector pairs and is obtained from the two ***k***-th coarse-grained time series at a scale factor of τ. Similarly, ${}^{m+1}n_{k}^{\tau }$ is the total number of matches of length m+1. Finally, the CMPCSE is calculated with the equation:

(8) $$\begin{array}{c} CMPCSE\left(x,y,z,\tau ,m,r\right)=CMCSE\left({\bar{r}}_{x},{\bar{r}}_{y},\tau ,m,r\right)\\ =\frac{1}{\tau^{*}}\sum_{k^{*}}CSE\left(u_{k^{*}}^{\tau },v_{k^{*}}^{\tau },m,r\right)=\frac{1}{\tau^{*}}\sum_{k^{*}}-ln\left(\frac{{}^{m+1}n_{k^{*}}^{\tau }}{{}^{m}n_{k^{*}}^{\tau }}\right), \end{array}$$

where $k^{*}$ means that neither ${}^{m+1}n_{k^{*}}^{\tau }$ nor ${}^{m}n_{k^{*}}^{\tau }$ is zero, that is, $-ln\left(\frac{{}^{m+1}n_{k^{*}}^{\tau }}{{}^{m}n_{k^{*}}^{\tau }}\right)$ makes sense, and $\tau^{*}$ is the number that makes $-ln\left(\frac{{}^{m+1}n_{k^{*}}^{\tau }}{{}^{m}n_{k^{*}}^{\tau }}\right)$ meaningful at a scale factor τ.

**Figure 3:**
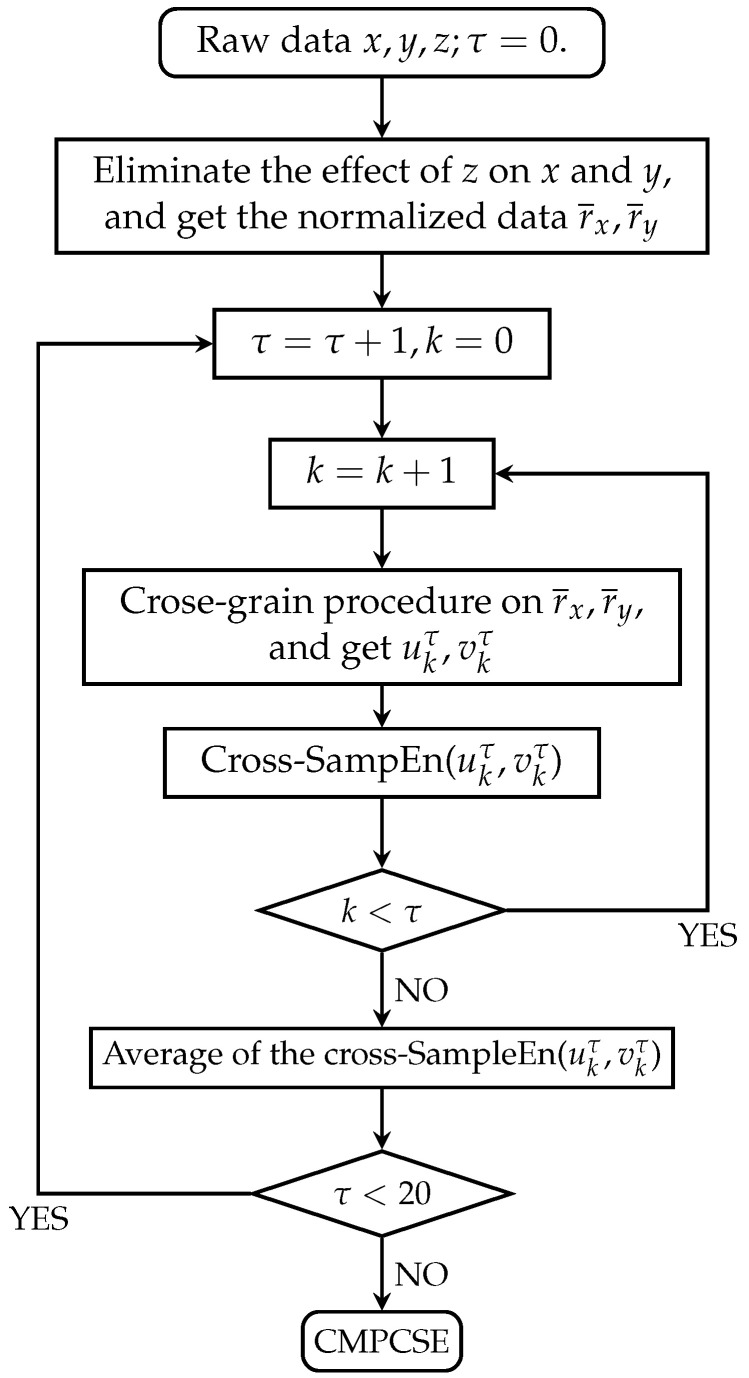
Flow charts of the CMPCSE algorithms.

A more intuitive procedure of CMPCSE is shown in Figure 3.

In this paper, the entropies are calculated from scale 1 to 20, that is τ=1,2,3,…,20. And the cross-sample entropy of each pair of coarse-grained series is calculated with m=2 and $r=r^{*}$, where $r^{*}$ is the value selected from the candidate set {0.05,0.1,0.15,…,0.95} according to the criterion proposed by Lake et al. [16].

## 3. Numerical Experiments for Artificial Time Series

In this section, we use a additive model of *x* and *y* as Equation (9) to perform numerical simulation and verify the effectiveness of the CMPCSE.

(9) $$\left\{\begin{array}{c} x\left(t\right)=2+3z\left(t\right)+r_{x}\left(t\right),\\ y\left(t\right)=2+3z\left(t\right)+r_{y}\left(t\right). \end{array}\right.$$

In the following numerical simulations, the series $r_{x}\left(t\right),r_{y}\left(t\right)$ are generated from the Bivariate Fractional Brownian Motion (BFBMs), TWO-component ARFIMA process and Multifractal binomial measures, respectively, and all the third party interference factor series z(t) are pink(1/f) noise generated by the *DSP System Toolbox* in MATLAB 2016. In the experiments, all the results about the sequences with random terms are the average of 100 repeated results with series length $N=2^{12}$.

### 3.1. Bivariate Fractional Brownian Motion (BFBMs)

In this subsection, in order to test the performance of CMPCSE, we first use it to calculate the partial cross-sample entropy of BFBMs in the two sets of the above additive models (Equation (9)). The $r_{x}$ and $r_{y}$ are the incremental series of the two components of BFBMs with Hurst indices $H_{r_{x}}$ and $H_{r_{y}}$. Extensive research on BFMS has been made. We know that BFBMs is a single fractal process and there is a relationship $H_{r_{x}r_{y}}=\left(H_{r_{x}}+H_{r_{y}}\right)/2$ [41,42,43]. Wei et al. studied the long-range power cross-correlations between $r_{x}$ and $r_{y}$ in 2017 [40]. In the simulations, we set: (left) $H_{r_{x}}=0.6$, $H_{r_{y}}=0.7$, ρ=0.7; (right) $H_{r_{x}}=0.6,H_{r_{y}}=0.9,\rho =0.7$; where ρ is the cross-correlation coefficient between $r_{x}$ and $r_{y}$.

**Figure 4:**
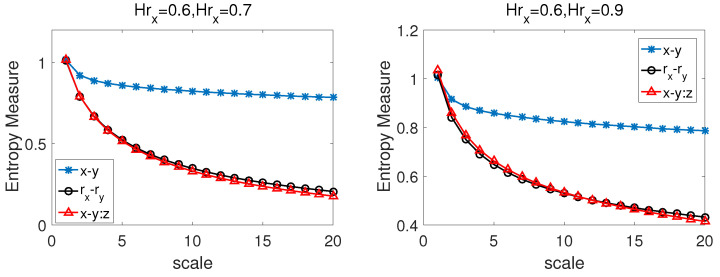
The CMPCSE results between the series simulated by the pink noise and bivariate fractional Brownian motion (BFBMs) with (**left**) $H_{r_{x}}=0.6,H_{r_{y}}=0.7,\rho =0.7$; (**right**) $H_{r_{x}}=0.6,H_{r_{y}}=0.9,\rho =0.7.$

We apply the CMPCSE method to the series simulated by BFBMs and pink noise. Figure 4 shows the results between the series simulated by the pink noise and BFBMs with (left) $H_{r_{x}}=0.6$, $H_{r_{y}}=0.7$, ρ=0.7; (right) $H_{r_{x}}=0.6,H_{r_{y}}=0.9,\rho =0.7.$ From Figure 4 we can know that the entropy values of x−y:z and $r_{x}-r_{y}$ are very close at all time scales, but there are obviously discrepancy between the values of x−y:z and x−y except when the time scale equal to 1, which indicates that, when $r_{x},r_{y}$ are affected by the third party factor *z* simultaneously, the CMPCSE method can capture the intrinsic cross-sample entropy values of $r_{x},r_{y}$ by eliminating the influence of *z*.

### 3.2. TWO-Component ARFIMA Process

ARFIMA process is a monofractal process [40] and often used to model the power-law auto-correlations in stochastic variables [44]. It is defined as follows:

(10) $$g\left(t\right)=G\left(t\right)+\varepsilon_{g}\left(t\right),$$

where d∈(0,0.5) is a memory parameter, $\varepsilon_{g}$ is an independent and identically distributed Gaussian variable, and $G\left(d,t\right)=\sum_{n=1}^{\infty }a_{n}\left(d\right)g\left(t-n\right)$, in which $a_{n}\left(d\right)$ is the weight $a_{n}\left(d\right)=d\Gamma \left(n-d\right)/\left[\Gamma \left(1-d\right)\Gamma \left(n+1\right)\right]$. The Hurst index $H_{GG}$ is related to the memory parameters [45,46]. For the two-component ARFIMA processes discussed below, we take G=X or *Y*. The two-component ARFIMA process is defined as follows [47]:

(11) $$\left\{\begin{array}{c} r_{x}\left(t\right)=WX\left(d_{1},t\right)+\left(1-W\right)Y\left(d_{2},t\right)+\varepsilon_{r_{x}}\left(t\right),\\ r_{y}\left(t\right)=\left(1-W\right)X\left(d_{1},t\right)+\left(1-W\right)Y\left(d_{2},t\right)+\varepsilon_{r_{y}}\left(t\right), \end{array}\right.$$

where W∈[0.5,1] quantifies the coupling strength between the two processes $r_{x}\left(t\right)$ and $r_{y}\left(t\right)$. When W=1, $r_{x}\left(t\right)$ and $r_{y}\left(t\right)$ are fully decoupled and become two separate ARFIMA processes as defined in Equation (11). The cross-correlation between $r_{x}\left(t\right)$ and $r_{y}\left(t\right)$ increases when *W* decreases from 1 to 0.5 [47].

In the process of our calculations, we choose W=0.8 and the parameters $\left(d_{1},d_{2}\right)$ of ARFIMA as $d_{1}=0.1,d_{2}=0.2$ and $d_{1}=0.1,d_{2}=0.4$ respectively, and corresponding two error terms $\varepsilon_{r_{x}}\left(t\right)$ and $\varepsilon_{r_{y}}\left(t\right)$ share one independent and identically distributed Gaussian variable with zero mean and unit variance. The CMPCSE method was used to the series simulated by two-component ARFIMA process and pink noise.

**Figure 5:**
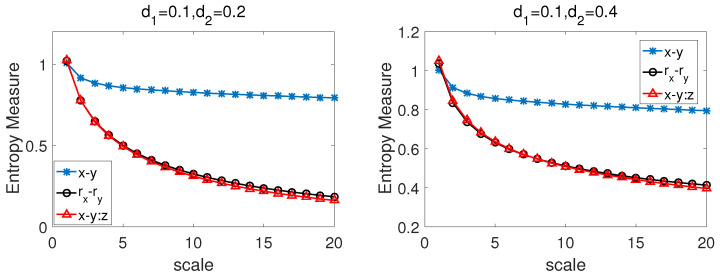
The CMPCSE results between the series simulated by the pink noise and two-component ARFIMA process with (**left**) $d_{1}=0.1,d_{2}=0.2,W=0.8$; (**right**) $d_{1}=0.1,d_{2}=0.4,W=0.8.$

Figure 5 also shows that the entropy values of x−y:z and $r_{x}-r_{y}$ are very close at all time scales, but there are obviously discrepancy between the values of x−y:z and x−y except when the time scale equal 1. It also means that, when $r_{x},r_{y}$ are affected by the third party factor *z* simultaneously, one can use the CMPCSE to get intrinsic cross-sample entropy values of $r_{x},r_{y}$.

### 3.3. Multifractal Binomial Measures

In this subsection, the series $r_{x},r_{y}$ to be tested come from the binomial measures generated by p−model with known analytic multifractal properties [40]. We combine them with pink noise to test the performance of CMPCSE. Each binomial measure or multifractal signal can be generated by iteration. We start with the iteration k=0, where the data set g(i) consists of one value, $g^{\left(0\right)}\left(1\right)=1$. In the *k*th iteration, the data set $\left\{g^{\left(k\right)}\left(i\right),i=1,2,\ldots ,z^{k}\right\}$ is obtained from $g^{\left(k\right)}\left(2i-1\right)=pg^{\left(k-1\right)}\left(i\right)$ and $g^{\left(k\right)}\left(2i\right)=\left(1-p\right)g^{\left(k-1\right)}\left(i\right)$. When $k\to \infty ,\;g^{\left(k\right)}\left(i\right)$ approaches to a binomial measures, and the scaling exponent function $H_{gg}\left(q\right)$ is:

(12) $$H_{gg}\left(q\right)=1/q-log_{2}\left[p^{q}+{\left(1-p\right)}^{q}\right]/q.$$

In our simulation, we iterated 12 times with $p_{1}=0.2,p_{2}=0.3,p_{3}=0.4$ and then get 3 binomial measures $g_{p_{1}}\left(i\right),g_{p_{2}}\left(i\right),g_{p_{3}}\left(i\right)$. In our actual calculation process, we set $r_{x}=$ *diff*$\left(g_{p}\left(i\right)\right)$, here *diff* means the first order difference.

**Figure 6:**
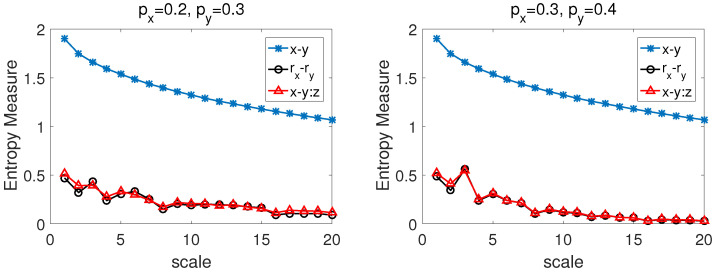
The CMPCSE results between the series simulated by the pink noise and first order difference series of the binomial measures (**left**) $p_{x}=0.2,p_{y}=0.3$; (**right**) $p_{x}=0.3,p_{y}=0.4$.

We present CMCSE results of the series x−y, $r_{x}-r_{y}$ and the CMPCSE x−y:z in Figure 6 with $p_{x}=0.2,\;p_{y}=0.3$ and $p_{x}=0.3,\;p_{y}=0.4$. From the two pictures in Figure 6, we can easily find out that the entropy values of x−y:z and $r_{x}-r_{y}$ are very close at all time scales, but there are obviously discrepancy between the values of x−y:z and x−y. It also indicates that, when $r_{x},r_{y}$ are affected by the third party factor *z* simultaneously, one can use the CMPCSE method to get intrinsic cross-sample entropy values of $r_{x},r_{y}$ by eliminating the influence of *z* on x,y.

## 4. Application to Stock Market Index

In order to validate the applicability of the CMPCSE method for empirical time series, we then apply it to stock market indices. The analyzed data sets consist of three Chinese stock indices: Shanghai securities composite index (SSEC), Shenzhen Stock Exchange Component Index (SZSE) and Hang Seng Index (HSI). All the raw data were download from https://finance.yahoo.com/. Then the daily closing data for the indices from 26 December 1999, to 17 July 2020, were used. Due to the different opening dates in mainland and Hong Kong, we exclude the data recorded on different dates and then reconnect the remaining parts of the original series to obtain time series with same length. As a result, the final daily closing data length is 5000.

In practice, we usually apply normalized time series. Denoting the closing index on the *t*th days as x(t), the daily index return is defined by: g(t)=ln(x(t))−ln(x(t−1)). Then the normalized daily return is defined as R(t)=(g(t)−<g(t)>)/δ, where <g(t)> and δ are the mean value and standard deviation of the series g(t), respectively.

**Figure 7:**
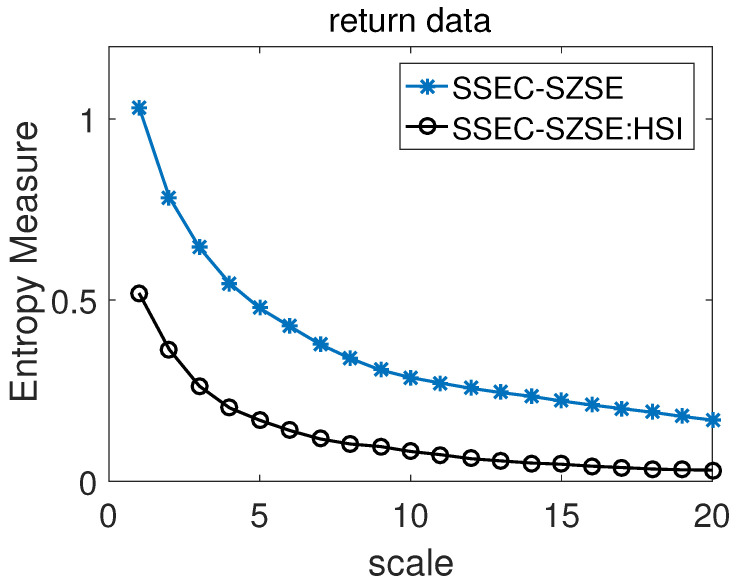
Estimation and comparison of the cross-sample entropy between the two return time series Shanghai securities composite index (SSEC) and Shenzhen Stock Exchange Component Index (SZSE) when including and excluding the influence of the Hang Seng Index (HSI) index.

In 2015, Shi and Shang studied the multisacle cross-correlation coefficient and multisacle cross-sample entropy between SSEC, SZSE and HSI [48]. From their results, we can know that there is a strong correlation between the return data of SSEC and SZSE, and both them have weak correlation with HSI. The results of our estimation and comparison of the cross-sample entropy of the two return time series SSEC and SZSE, which includes two cases of including and excluding the influence of the HSI index, are shown in Figure 7. From the entropy measure results of return data in Figure 7, one can easily find that the entropy values of SSEC-SZSE are always bigger than SSEC-SZSE:HSI at all scales, which means that if the entropy values of SSEC-SZSE calculated by CMCSE are used to estimate the degree of similarity between SSEC and SZSE, the similarity between them will be underestimated. That is to say, the partial cross-sample entropy SSEC-SZSE:HSI can deliver more reasonable and real synchronization between the two return time series of SSEC and SZSE. We believe this result is reasonable, as SSEC and SZSE are the two most important stock indices in the mainland of china, so their daily return data should have strong synchronicity, especially under large time scales.

## 5. Discussion and Conclusions

In this paper, we proposed CMPCSE for quantifying intrinsic similarity of two time series affected by common external factors. Firstly, we described the calculation process of CMPCSE in detail. And then, in order to test the validity of CMPCSE, we applied it to three sets of artificial data. These three sets of artificial data were constructed by linear superposition of BFBMs, TWO-component ARFIMA process and Multifractal binomial measures with pink(1/f) noise respectively. The results of each set of the artificial data show that CMPCSE can accurately measure the intrinsic cross-sample entropy of two simultaneously recorded time series by removing the effects that come from pink noise. At last, CMPCSE was employed to investigate the partial cross-sample entropy of SSEC and SZSE by eliminating the effect of HSI. Compared with the conclusion from CMCSE, the results from CMPCSE show that SSEC and SZSE have stronger similarity. Because SSEC and SZSE are the two most important stock indices in the mainland of China, they should have strong consistency, especially under large time scales, so we think the result is reasonable and it is necessary to consider partial cross-sample entropy when one wants to measure the similarity of SZSE and SSEC.

On the other hand, we must also note that the first step in the calculation of CMPCSE is crucial to the result of CMPCSE. Maybe there are other ways to eliminate the influence of the third party on the two time series that we studied. In our work, we adopted the idea from Reference [40] and satisfactory results were obtained in our artificial data examples. At the same time, in our research process, we also notice that when CMPCSE is used to study the linear combination of NBVP times series mentioned in Reference [26] and pink noise, which is constructed in the way mentioned above, we can not get satisfactory results. Therefore, we think that the way to eliminate the third-party influence in this paper can not achieve good results for the sequence with violent oscillation. Meanwhile, we expect to see better methods to deal with similar times series.

All in all, we think the partial cross-sample entropy analysis is necessary when one wants to measure the similarity of two times series affected by common external factors and, at present, CMPCSE is a good choice.
